# Terahertz spectroscopy analysis of human corneal sublayers

**DOI:** 10.1117/1.JBO.26.4.043011

**Published:** 2021-04-26

**Authors:** Lin Ke, Qing Yang Steve Wu, Nan Zhang, Zaifeng Yang, Erica Pei Wen Teo, Jodhbir S. Mehta, Yu-Chi Liu

**Affiliations:** a Institute of Materials Research and Engineering, Agency for Science, Technology and Research, Singapore; b Institute of High Performance Computing, Agency for Science, Technology and Research, Singapore; c Singapore Eye Research Institute, Singapore; d Singapore National Eye Centre, Singapore; e Duke-NUS Medical School, Ophthalmology and Visual Science Academic Clinical Research Program, Singapore

**Keywords:** cornea, edema, terahertz, spectroscopy, hydration, noninvasive

## Abstract

**Significance:** Corneal diseases is a major cause of reversible blindness in the world. Monitoring the progression of human corneal edema or corneal scarring to prevent the disease entering into the end stage is crucial.

**Aim:** We present a method for sensing human corneal composition at different depths, namely focused on the epithelium and stromal layer, using high-sensitivity terahertz (THz) broadband spectroscopy.

**Approach:** From the proposed methodology, the THz temporal and absorption spectra of human corneas at different edema stages have been studied. THz wave signals were collected from the direct reflection and four other collection points along the THz wave propagation direction as reviewed from the simulation THz electrical field.

**Result:** Our results show that the epithelium layer acts as a good barrier to maintain hydration level of the stroma, and the quality of the epithelium can be used to predict the level of corneal swelling in corneal edema. At the detection points near to the incident point, the THz frequency spectra demonstrated interference oscillation behavior. At the final edema observing time, results showed that the epithelium lose its barrier properties. The intactness of the epithelium can be used to predict the edema severity in the final stage. When the detection points are further away from the incident point, the THz spectra are believed to contain information from stromal layer. Stromal absorption spectra demonstrated correlation with optical coherence tomography thickness results.

**Conclusion:** The hydration concentration from stromal layer was further quantitatively calculated. At the end of the experiment, all the corneal hydration levels reach to the same value which shows that the edema hydration has reached maximum saturation. The information of individual sublayers of the cornea is obtained by characterizing noninvasively with the use of THz spectroscopy. To our knowledge, this is the first report of using THz for noninvasive characterization of sublayers of the cornea.

## 1 Introduction

Corneal diseases are the major causes of irreversible blindness in the world. The hydration level of cornea is essential in maintaining the transparency and hydrodynamics function of corneal.^1^ It also plays a crucial part in a variety of corneal conditions, such as corneal inflammation, corneal trauma, or corneal edema. Moreover, it is known that corneal hydration can affect the efficacy and safety outcomes of refractive surgery.^2^^–^^5^ Hence, tools and methods to more precisely evaluate and monitor the hydration level in corneas would be useful. However, there are no available tools to accurately evaluate corneal hydration directly so far. Corneal thickness, measured by either ultrasonic pachymeter or optical coherence tomography (OCT), is used by ophthalmologists as surrogate measures and indirect evaluation of corneal hydration.^6^ Corneal hydration is currently approximated in the clinic by extrapolation using the central corneal thickness (CCT) measurements. Deviations of 20% in CCT or greater are observed in the data, and the extrapolation method is based upon healthy corneas. Therefore, it cannot provide accurate analysis for changes relating to disease states in corneas.^7^^–^^9^

Terahertz (THz) detection is a nondestructive, noninvasive, noncontact, and biologically safe measurement with fast scanning speed to meet the medical diagnostic requirements. Wilmink et al.^10^ and our group^11^ demonstrated the safety profiles of THz scanning in ophthalmology at a tissue, cellular, structural, and functional level. THz time of flight measurement gives more accurate results for the estimation of the corneal thickness. Its high sensitivity hydration absorption and characterization peaks of collagen provide a useful tool to obtain information related to corneal layers.^12^^–^^15^ Researchers from the University of California Los Angeles have been working on THz *in vivo* hydration evaluation in the past 10 years.^16^^–^^19^ Their latest work focused on the simulation of THz spectral properties of human cornea as a function of CCT and corneal water content. Based on corneal physiology, different factors affecting corneal tissue water content were selected as perturbations, and systematic simulation for the effects on the water distribution and total thickness have been done. Liu et al.^20^ detected the optical properties of *ex vivo* rabbit corneal tissues with different water content at THz frequencies and discovered that the absorption coefficient and refractive index of a hydrated cornea were much larger than that of a dehydrated cornea. Recently, researchers from Lomonosov Moscow State University^21^^–^^24^ developed THz simplified dielectric permittivity model of water and water-containing media by considering cornea as a homogeneous medium, compared with relatively larger wavelength in THz range, and quantitative calculation of the physiological dynamics of tear film and hydration were reported. The penetration depth of THz waves and the sensitivity of the reflected component to the dynamics of evaporation of the tear film were discussed. The group further reported the reflection coefficient of the ocular surface extracted using continuous THz photomixer reflectometers from *in vivo* experiments.

However, there is still an unmet gap for THz technology to be applied in clinical applications. For example, current results are limited to assess the hydration of the surface of corneal or tear film rather than on individual deep corneal layers. It was not possible to assess the epithelium/stroma/endothelium interfaces, hydration distribution the cornea, and other corneal components concentration across the depth. In this work, THz time-domain spectroscopy (TDS) was applied to investigate corneal sublayer hydrodynamic behaviors, namely epithelium and stromal layer, to give a clear picture across the corneal depth.

## 2 Materials and Methods

### 2.1 Materials

A total of three batches of 15 human cadaveric corneas were procured from overseas eye banks (Lions Eye Bank, Rochester, New York) after donors’ consents being taken. These corneas had different death-to-preservation time and different preservation times before arrival hence presented with different extent of corneal edema. These corneas were scanned at days 1, 4, 8, 12, 18, 25, 30, 33, and 37.

### 2.2 THz Beam Simulation

Noninvasive quantitative measurement of the concentration of different absorbing substances in living body tissue such as cornea is challenging. The incident THz beam could travel along the sublayers with path losses due to the spreading of the propagating wave, absorption from different types of molecules as well as scattering from both the cells and the medium water background. THz propagation in corneas is determined by many factors, such as incident geometric structure, THz wavelength variations, optical properties of the media including absorption coefficients, and reduced scattering coefficient and scattering angle distribution, etc.

**Fig. 1:**
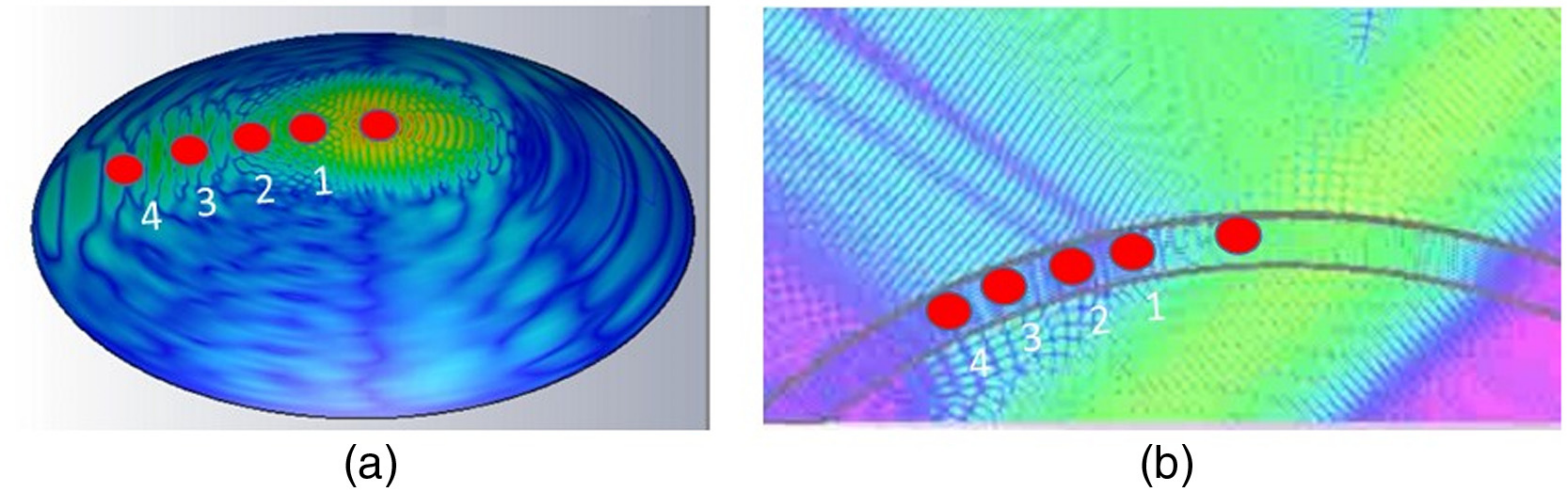
(a) Simulated electrical field on the cornea. (b) The cross view of the simulated electric field distribution.

Simulation of THz beam path inside the corneal sample has been implemented using CST Studio Suite: electromagnetic field simulation software. The simulated THz beam power is 1 W at 1 THz, linear polarized, the distance from source plane to the focal plane is 6 mm, and the beam waist is 1 mm. The relative permittivity for epithelium, stromal, and bottom layers are 4.5, 6, and 4.5, with thickness of 50, 700, and 30  μm, respectively. The incident angle is 30 deg and the THz waves are focused on the epithelium layer surface. Figure 1(a) shows the simulated electrical field distribution on the cornea. Figure 1(b) shows the cross view of how the THz beam propagates inside the cornea structure. As we observed from the simulated results, the THz waves can penetrate the three-layer modeled cornea.

The simulated results show that when the beam is incident on the surface of the cornea, significant part of the wave will pass through the cornea. Certain amount of the THz waves will be directly reflected according to the Snell’s Law. If the THz beam is incident on the corneal surface with an angle, the waves will propagate mainly in one direction at the incident point as shown in Fig. 1 (higher electrical field indicated by orange and green color). The simulated electromagnetic field demonstrated that there are waves going through much longer propagation distance along the corneal internal structures. These reflected waves could bring more information from the deeper layer of the corneas to the detector. Therefore, in our experiments, we position the detector closer to the cornea surface and moved away from the direct reflection position of 30 deg and collected series of signals at points 1, 2, 3, and 4, as shown in Fig. 1(a) by red dots.

### 2.3 THz Spectroscopy Edema Analysis Experimental Setup

The THz spectroscopy system contains femtosecond fiber lasers with 250-MHz repetition rate and 90 femtosecond laser pulses with around 1.56-μm central wavelength, which are used for two photoconductive antennas: one for emitter and another one for receiver. The THz pulse is generated with the coverage of bandwidth up to 3 THz.

**Fig. 2:**
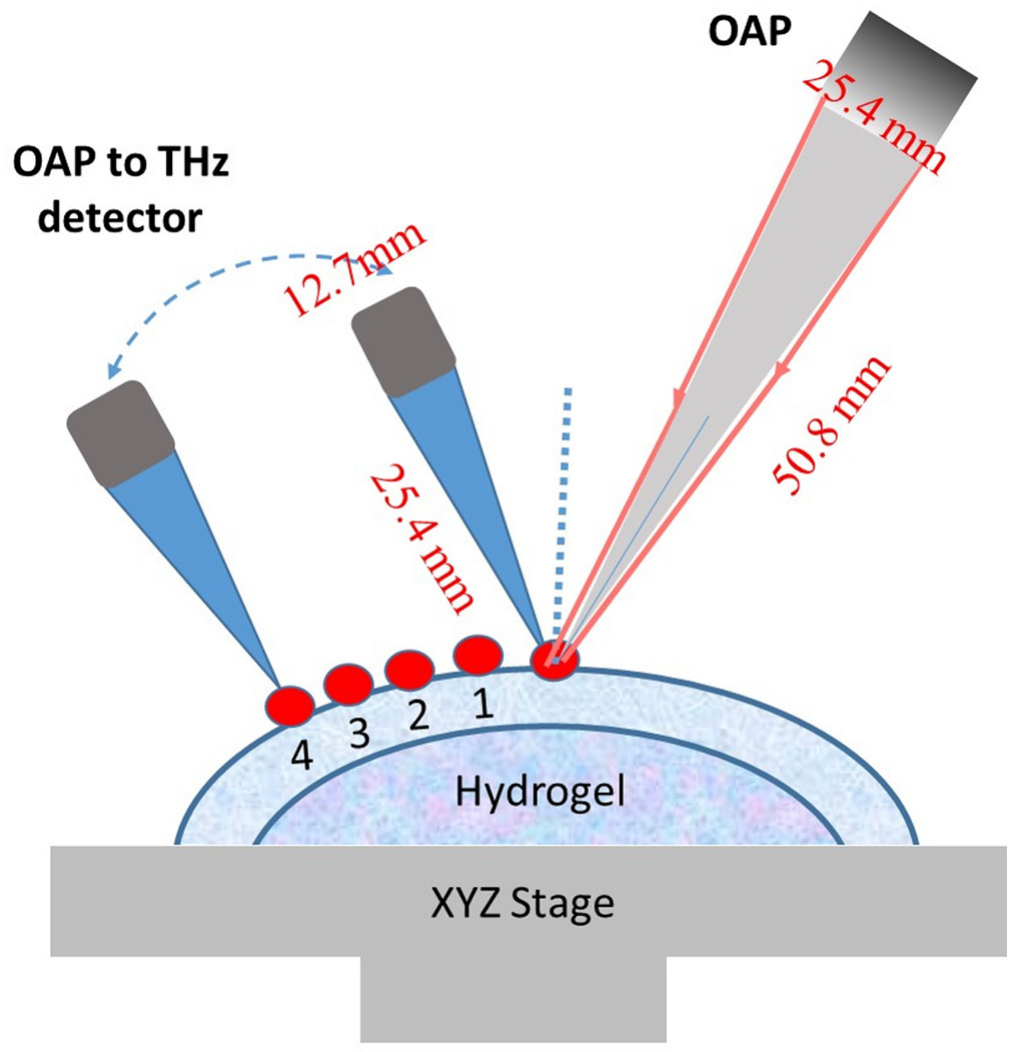
The schematic diagram of the THz signal collected from the corneal surface and corneal subsurface.

THz source beam is collimated by a 76.2-mm effective focal length (EFL), 25.4-mm clear aperture off-axis 90-deg parabolic (OAP) mirror. A 50.8-mm EFL OAP mirror at a 30-deg incidence angle is used to focus the beam onto the target. The reflected THz radiation is then collected and collimated by a 12.7-mm diameter, 25.4-mm EFL. The system optical layout is shown in Fig. 2.

At first, the beam from the emitter focuses on the cornea far front surface through parabolic mirrors as indicated in the figure. Focusing has been fine-tuned to achieve the highest TDS intensity. One single shot of THz field waveform was collected with incident angle of 30 deg. Thereafter, the detector moved away from the direct reflection position and collected a series of reflected beams continuously at different distances away from the direct reflections denoted as points 1, 2, 3, and 4 as indicated by Figs. 1(a) and 2. At each point, the focus has been fine-tuned to achieve the highest TDS intensity. The distance between each point is 2 mm.

### 2.4 Absorption Spectra Analysis

In THz reflection geometry, the calculations of the absorption spectra and the extraction of dielectric properties (index of refraction and absorption coefficient) from THz-TDS measurements rely profoundly on the retrieval of the optical phase of the THz wave. To achieve a qualitative measurement of the absorption behavior from the cornea sublayers, our THz system was calibrated by maximizing the reflectivity off a strip of aluminum. The cut thin corneal sample was placed on aluminum stage with hydrogel filled in behind the thin corneal layer to mimic the real eyeball. To extract the cornea property parameters, a numerical fitting technique is used. This technique first uses a theoretical model that mimics the corneal multilayers and hydrogel layer behind to simulate the reflected spectrum. An optimization algorithm compares the simulated reflected spectrum to the experimental result. The difference between them can be minimized by selectively altering the parameters in the theoretical model. The parameters with the best fit will give the refractive index of the samples. Thus, the absorption spectra can be further calculated.

### 2.5 Optical Coherence and Thickness Measurement

After scanning by the THz spectroscopy, the corneas were immediately scanned by the RTVue ASOCT (Optovue, Inc., Fremont). Three high-resolution corneal cross-sectional scans (8-mm scan length, single scan mode) were obtained for each sample at each time instant. The CCT was measured using the built-in software calipers at the corneal center by a single observer.^25^ The measurements obtained from the THz system and ASOCT were compared.

## 3 Results and Discussion

### 3.1 Time-Domain Analysis

**Fig. 3:**
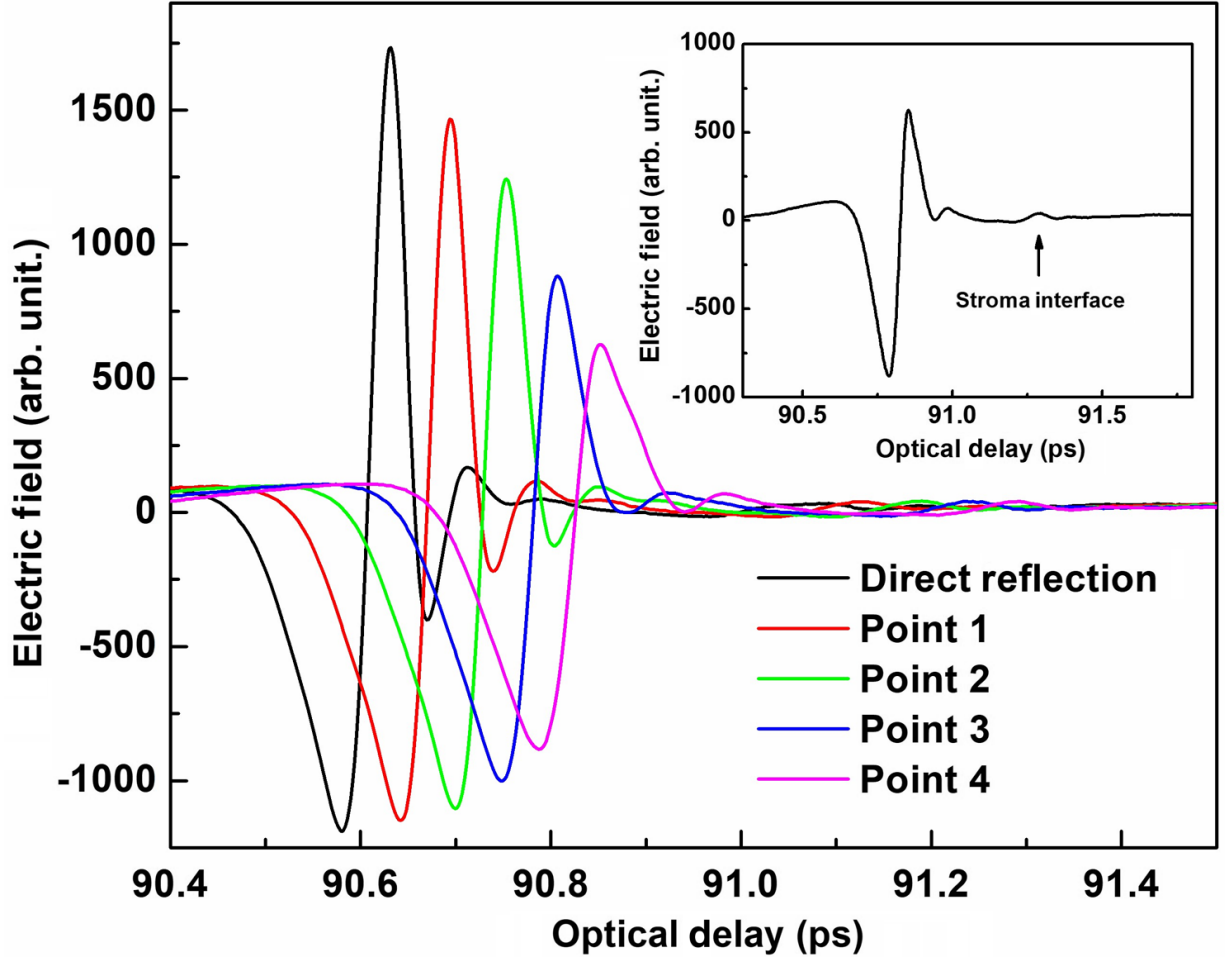
THz time-domain signals collected at the direct reflection at points 1, 2, 3, and 4, respectively.

The incident beam was focused on the epithelium far front surface at first. The focus has been fine-tuned to achieve the highest TDS intensity. THz field waveform was collected using an incident angle of 30 deg. Thereafter, the detector moved away from the direct reflection position and collected a series of reflection THz field waveforms at points 1, 2, 3, and 4. Figure 3 shows the THz time-domain signals collected from direct reflection and points 1, 2, 3, and 4 respectively. As shown in the enlarged inset figure in Fig. 3, the second reflection peaks were observed in time-domain waveform for direct reflection, which may indicate the epithelium/stroma interface, with signal–noise ratio of the TDS further improvement, deeper layers, and their interfaces could be further reviewed.

### 3.2 Absorption Spectra Analysis

**Fig. 4:**
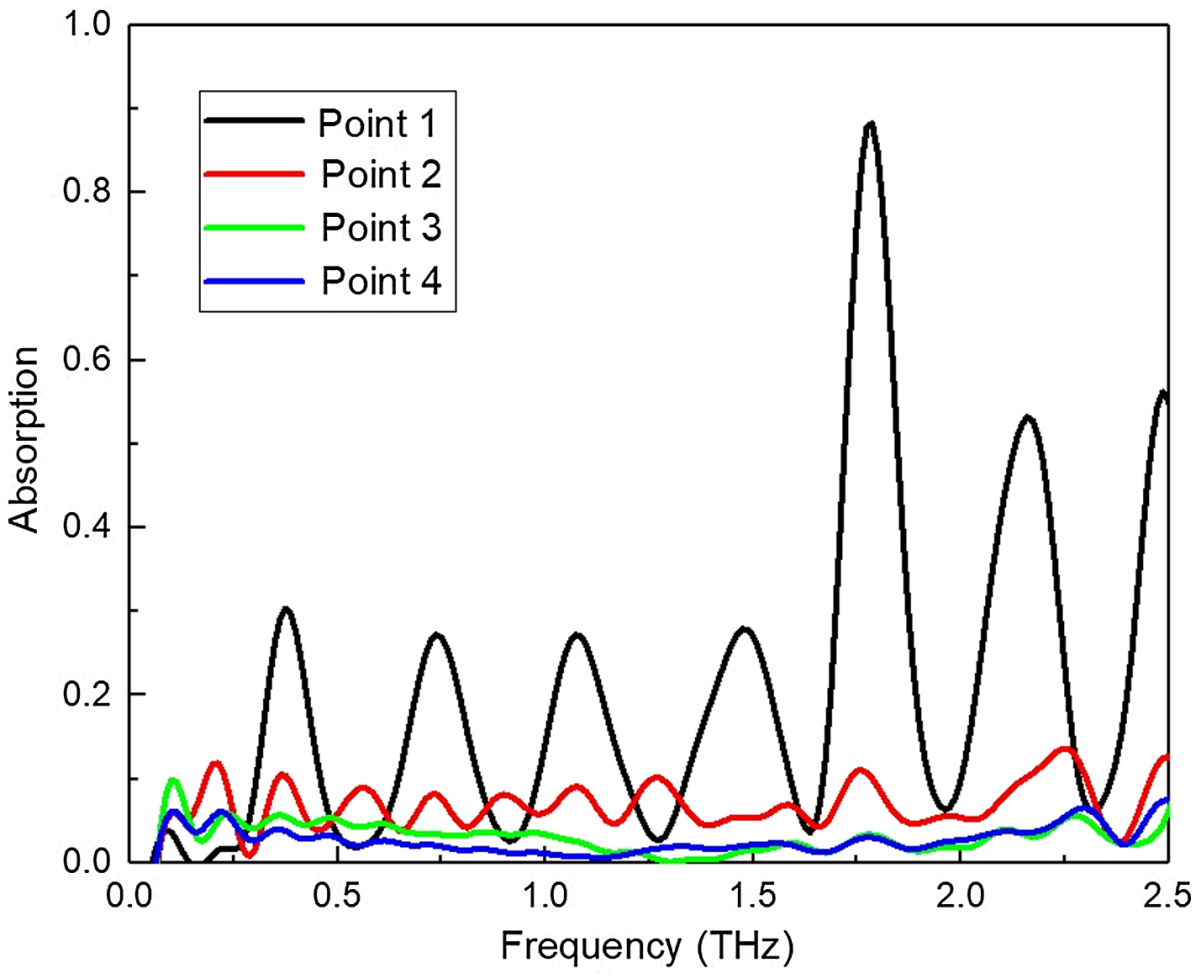
Absorption spectra calculated for the four targeted points 1, 2, 3, and 4, respectively.

Figure 4 shows the absorption spectra calculated based on the time-domain field waves collected from points of 1, 2, 3, to 4. For the point 1 absorption spectra, there are strong periodic interference fringes observed. At point 1, a slight deviation from the direct reflection, most of the THz beam travels inside the epithelium layer. The interference fringes are mainly caused by the reflected waves on the upper surface of epithelium and the interface between epithelium and stromal. The incident angle for THz beam to the epithelium surface directly caused the interference fringes. The interference oscillation periods are related to the thickness of the epithelium layer and the incident angles. Although such interference fringes could have a serious impact on the stromal information we need to extract, they can review the epithelium and epithelium/stromal interface performance. The oscillation peak magnitude and phase could review the epithelium and interface property. The stronger peak reviews the better and distinct interface between the epithelium and stroma.^27^^,^^28^ Multiple optical interference effects in thin layer structure can be used for the explanation of reflectance spectra performance of obvious interface fringe phenomenon. Based on the principles of interference theory, the thickness of the thin film can be calculated as

$$d=\frac{\Delta m}{2\sqrt{n^{2}-\sin^{2}\;\alpha }}x\frac{1}{\left(\frac{1}{\lambda_{2}}-\frac{1}{\lambda_{1}}\right)},$$ (1)

where “d” is the film thickness and “Δm” is the number of peaks in the wavelength range used for calculation.

In our experimental scheme, the incident beam has small incident angle, so sinα is small enough to be negligible. There are two maximum peaks at frequencies of $f_{1}=0.38$ and $f_{1}=0.74\;\mathrm{THz}$ in the Fig. 4. The high hydration level biology samples have dispersive refractive index behaviors in THz range, therefore the above equation can be further written as

$$d=\frac{M\lambda_{1}\lambda_{2}}{2(n_{\lambda 1}\lambda_{1}-n_{\lambda 2}\lambda_{2})}.$$ (2)

For high hydration biology samples, we adopted $n_{\lambda 1}=4$ and $n_{\lambda 2}=2.2$ from the reference which are corresponding to $f_{1}$ and $f_{2}$.^29^ Therefore, the estimated corresponding thickness is 68  μm. The calculated value is slightly higher than the healthy live cornea, the reason could be the corneal samples were purchased from overseas, and certain degree of edema has being developed during the transportation and storage.

For point 2, the apparent interference fringe peaks are still observed when the detection point is away from the direct reflection point due to that the wave could travel longer to reach the detector. When the detection reaches point 3 and point 4, the oscillation peaks were hardly observed. The information from point 3 and point 4 could reveal the stromal layer information. There are no significant absorption peaks at the stromal layer, which shows that the majority of the composition of the stromal layer is water.

### 3.3 Edema Progress Analysis

**Fig. 5:**
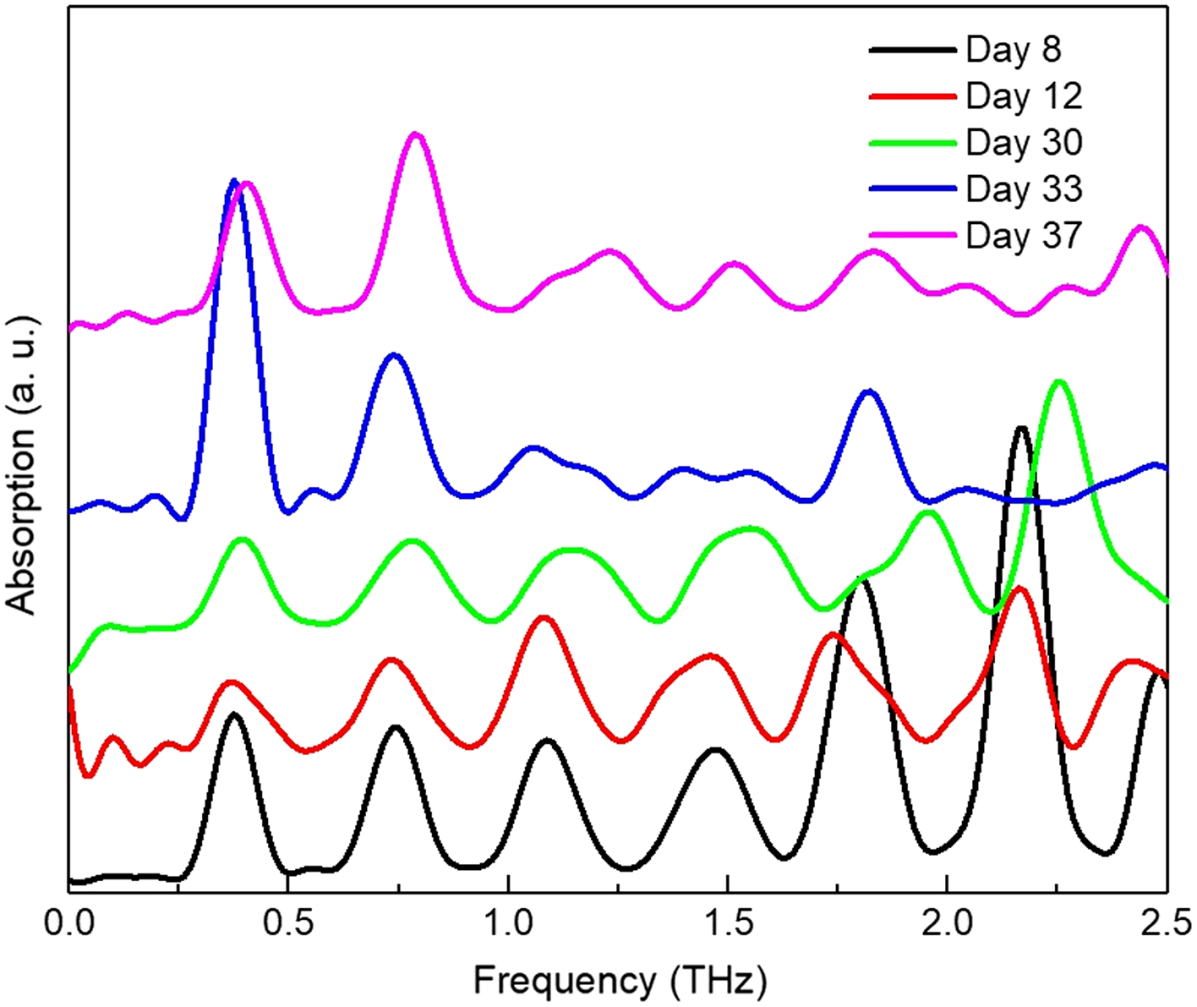
The absorption spectra calculated for one typical cornea at different edema levels at observation day of 8, 12, 30, 33, and 37, respectively.

We further observed the epithelium THz absorption spectral changes during edema formation. Figure 5 shows the representative absorption spectrum obtained for corneas with different severity of edema at point 1 until the final observation at 37 days. Curves for days 12, 30, 33, and 37 have been shifted down in the graph for better presentation. It shows that with the increasing of the corneal swelling, the oscillation peaks collected from point 1 will be slowly reduced. This could indicate that the epithelium/stromal interface became less distinctive, the epithelium slowly lost its structures and firmness. At the end of the observation, the corneal surface became much more swollen and opaque. The epithelium acts as the barrier from the environment for the stroma. The oscillation magnitude and period could have correlated to epithelium’s barrier capabilities and the quality.

### 3.4 Correlation Analysis

**Fig. 6:**
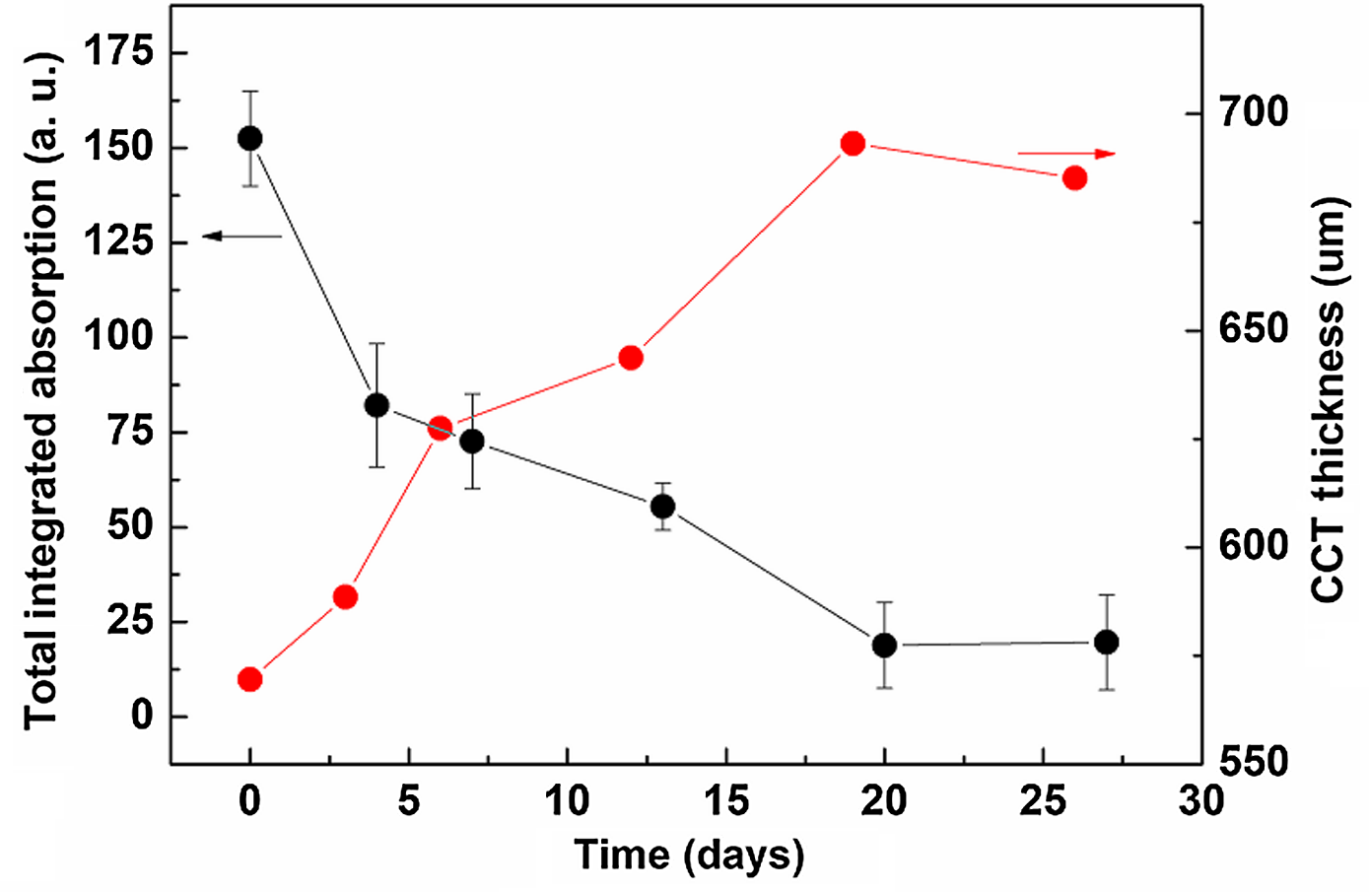
Total integration of absorption and thickness by CCT versus the observing days; black-colored dots: total integration of absorption; red-colored dots: cornea thickness.

Since the THz spectra collected from point 3 and point 4 indicate the stromal properties, there are hardly any absorption peaks can be observed. For the stromal layer, hydration is the main component. Therefore, the total integration method has been applied to further process the data in order to find the information related to hydration level. Figure 6 shows the total absorption integrated under the frequency of 0.1 to 2.5 THz versus the observing days for a typical cornea at point 3. The thickness information corresponding to the THz detection time is also shown in the same diagram for comparison. With the edema getting severe, the thickness will be increased while the total absorption will be decreased.

### 3.5 Hydration Level Quantitative Analysis

**Fig. 7:**
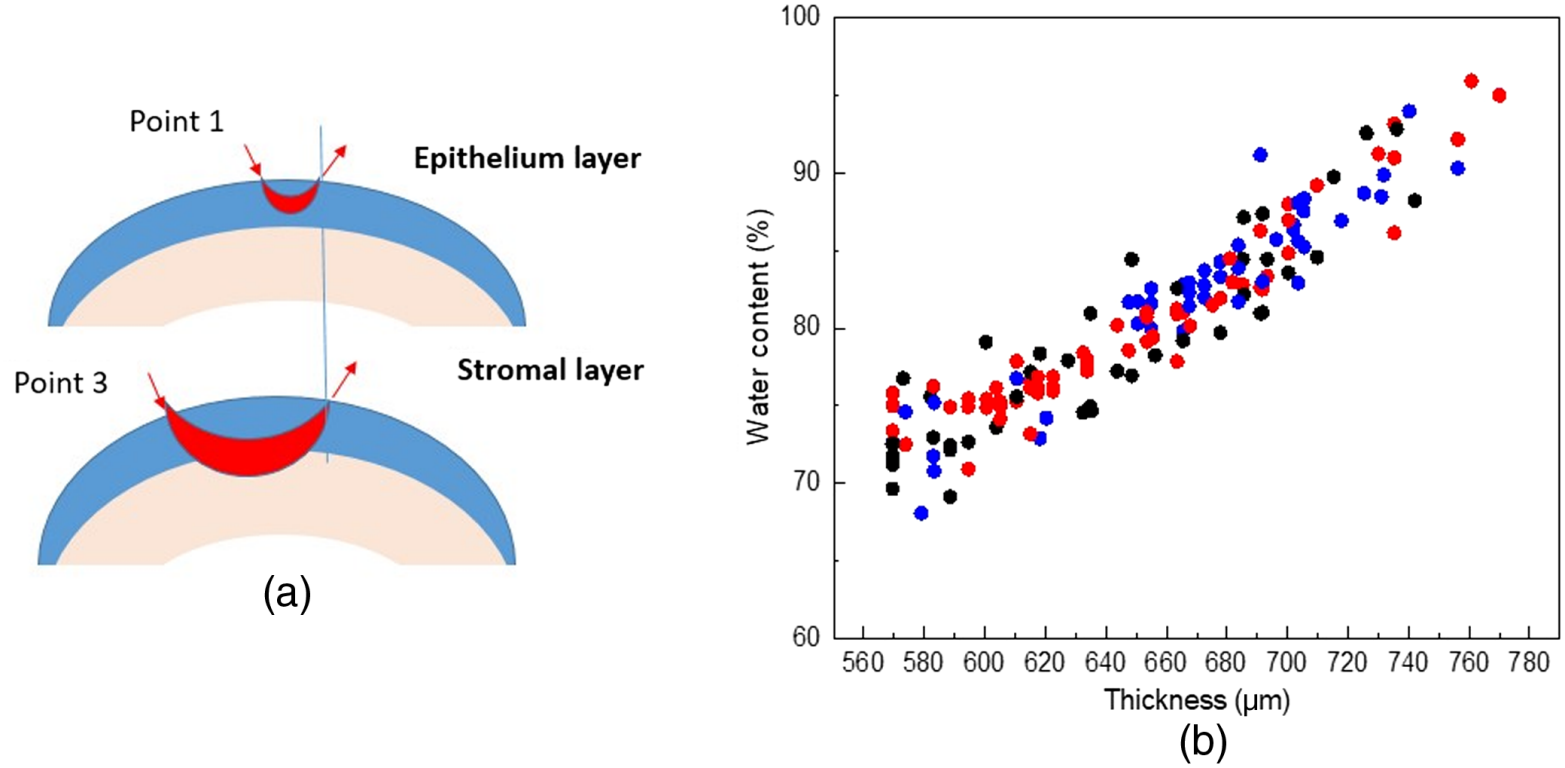
(a) THz wave path across the sublayer of the corneas. (b) The corneal water content calculated based on all three batch of 15 corneas versus thickness measured by CCT, black, blue, and red colored dots showed three different batches of cornea samples.

When we consider the depth target at point 3 and point 4, while the beam is traveling inside stromal layer. Figure 7(a) shows the THz wave path across the sublayer of the corneas. For point 1, when the waves cross over epithelium layer, oscillation peaks will be observed; when the waves cross over deeper to the stromal layer, less oscillation will be observed and information from the stroma could be carried out to the detector. Let the incident light be $I_{0}$ and the light of $I_{1}$ as detected intensity by the detector, the absorbance cross the wave path can be written as^30^^–^^32^

$$I(\nu {)}_{i}=I_{0}(\nu {)}_{i}\exp[k(\nu )]c_{i}l_{i},$$ (3)

where ν is the frequency of THz wave, $I_{0}(\nu {)}_{i}$ and $I(\nu {)}_{i}$ are the THz radiation intensities before and after penetrating the sample, k(ν) is the absorption of the sample, $c_{i}$ is the volume fraction of water, and $l_{i}$ is the thickness of the cube in the sample, $s_{i}$ is the length of the cube (the scanning step), and the volume of the cube is $s_{i}\times s_{i}\times l_{i}$. The water content in one cube can be calculated according to following equation:

$$M_{i}=\frac{s_{i}^{2}}{k(\nu )}\;\ln\;\frac{I_{0}(\nu {)}_{i}}{I(\nu {)}_{i}}.$$ (4)

The water content of the sample can be calculated by summing up all the cubes:

$$M=\sum M_{i}=\sum \frac{s_{i}^{2}}{k(\nu )}\;\ln\;\frac{I_{0}{\left(\nu \right)}_{i}}{I{\left(\nu \right)}_{i}}.$$ (5)

Figure 7(b) shows the corneal water content calculated based on all three batch of 15 corneas data. The thickness changed from 570  μm at the beginning of the observation to 750  μm at the end of the observation, and the calculated water content changes from 73% to 92%.

### 3.6 Prediction of the Final Degree of Corneal Edema

**Fig. 8:**
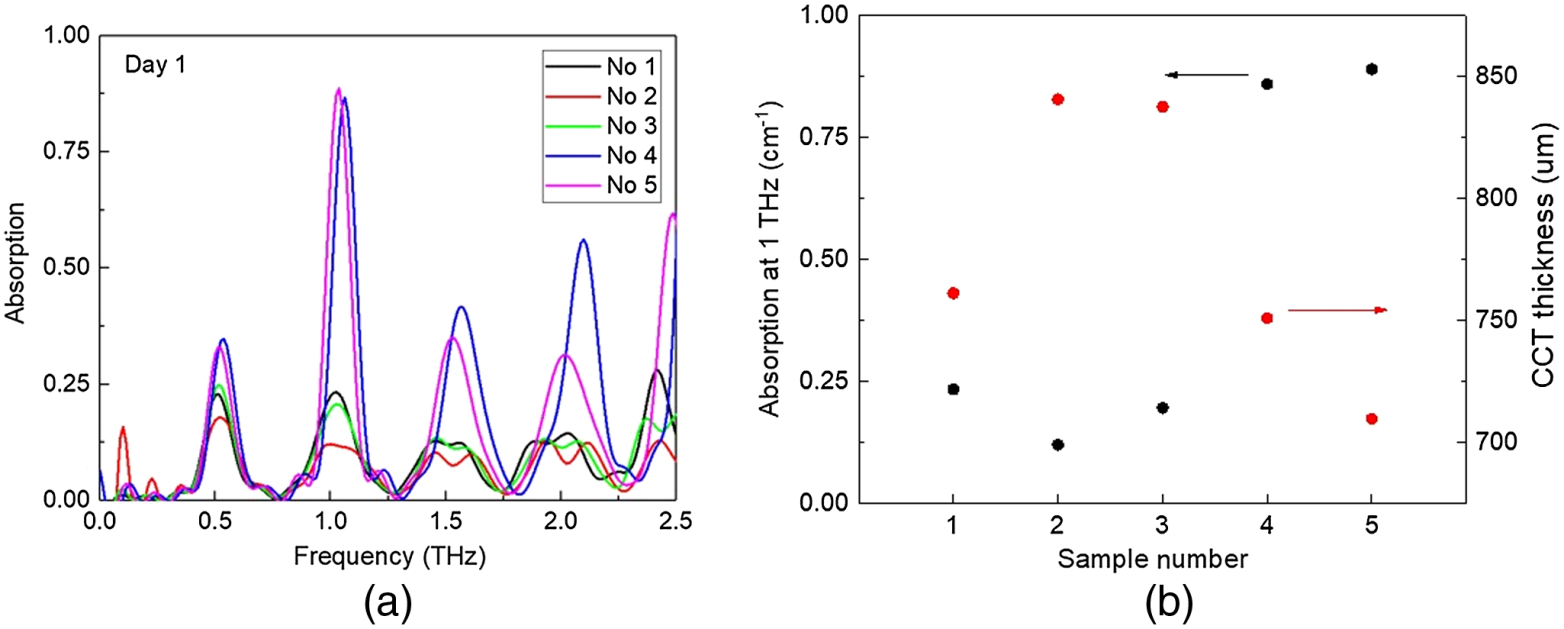
(a) Absorption spectra target at 50  μm depth at day 1 for five different corneal samples. (b) Absorption at 1 THz and CCT final thickness for the five corneal samples.

During each experiment, there are at least three measurements taken for each corneal sample. Good repeatability was observed. Figure 8(a) shows the first day of THz absorption spectra calculated based on the THz signal collected from point 1 position for one batch of five different corneal samples. The oscillation peak period is related to the thickness of the epithelium layer. Their differences were insignificant, on the contrary, the absorption intensity of the five corneas shows much more difference.

Corneas no. 2 and no. 3 show the smallest intensity while corneas no. 4 and no. 5 show the largest intensity. Good distinct oscillation peaks indicate that the no. 4 and no. 5 corneas had better epithelial property and good epithelium/stromal interfaces. We present the magnitude results at 1 THz for the five corneas measured on the first day of observation in Fig. 8(b). In the same diagram, the final corneal thickness measured on the last day of the observation period are also shown.

The results demonstrated that the initial stage of THz oscillation magnitude was in good agreement with the corneal end stage swollen thickness. The higher the initial oscillation magnitude, the clearer the epithelium interface, the better quality of the epithelium layer, the edema will be less severe, and the final stage swollen thickness will be smaller. From the evaluation of epithelium layer quality, we may be able to predict the final severity of the corneal edema from the beginning. The interface quality, uniformity, and thickness of the epithelium layer decided the oscillation peak magnitude and period. The good interface and smooth uniformed epithelium layer showed strong oscillation peaks. With a good epithelium layer, the stromal is not easy to be affected by external environment, therefore, the severity of edema will be reduced.

## 4 Conclusion

High-sensitivity THz broadband spectroscopy has been used for sensing corneal composition centering at different depths, namely the epithelium layer and stromal layer. THz wave propagated at the epithelial layer and showed oscillation behavior. The epithelium acts as a good barrier to prevent hydration increase in the stroma. The quality of the epithelium can be used to predict the level of swelling of the cornea in the later part of storage time. THz waves propagate through the stromal layer and carry information of hydration level. Total stromal absorption demonstrated a correlation with CCT results. Direct hydration value can be calculated, which avoids the need to choose standard samples for calibration of the hydration value. To our knowledge, this is the first report of using THz for noninvasive characterization of sublayers of the cornea.
